# News and Notes

**Published:** 1912-07

**Authors:** 


### NEWS AND NOTES

*Editor Journal-Record*:

I have not forgotten my promise to send you a communication from Berlin. In spite of the fact that the days here are so long that it does not get dark until almost ten P. M., "tempus fugit" very quickly.

There is nothing very remarkable to report; still there are a few impressions I have gained which may be of interest to some of your readers. There was a Surgical Congress held in Berlin and a Medical Congress at Weisbaden. Roentgenology seems to hold the attention of both the Medical and Surgical branches of the profession. Many papers on this subject were read and discussed at these meetings. The medical journals devote much space to Roentgenology and it looks as if the X-ray apparatus will eventually become as much of a necessity to a general practitioner as the microscope. At the surgical meeting the papers on Hormonal interested me the most.

Hormonal is a preparation made from the spleen and used by surgeons in post-operative intestinal paresis. It is injected intravenously and many surgeons report remarkable success with the substance, others admonish caution, as deaths have occurred in some instances.

It has been tried by many physicians in obstinate chronic constipation, one injection curing the patient, re-establishing intestinal peristalsis. Seems to have a similarity in some respects to Salvarsan, due to the remarkable effect of one injection and the comparative.

Salvarsan received a large share of attention at Weisbaden, the most interesting part being that quite a number reported the use of Salvarsan, especially the neo-salvarsan, in bad cases of scarlet fever with excellent results.

The Radium Therapy is sweeping all Europe. Every watering place the radium emanating virtues of its waters as baths and drinking cures, and its mud baths contain radium bromides.

Berlin is full of the so-called Radium Emanatoria.

I shall have more to say on this and other subjects on my return home.

The material at the Clinics and Polyclinics is so vast, the case one rarely sees at homes of almost daily occurrence here that one wishes to tarry longer.

A pleasant feature of the work is that the patients never get out of humor, and allow themselves to be studied and examined, even though it may cause them pain and inconvenience. They feel that they owe the profession a return for the treatment they receive.

To my satisfaction I observed that the German doctors have quite a different idea of American medicine and surgery than they had.

It is the custom for the more ambitious to go to America for a "Studenrreise," a trpp for study.

I have spoken to a number of men who have been in America and they are profuse in their praise of American scientific advance, its monumental institutions of learning and especially the charming American hospital of the medical profession.

L. AMSTER.

Berlin, June 1, 1912.

---

### AMERICAN PROCTOLOGIC SOCIETY.

### FOURTEENTH ANNUAL MEETING, HELD AT ATLANTIC CITY, N. J., JUNE 3 and 4, 1912.

The President, Dr. John L. Jelks, of Memphis, Tenn., in the chair.

Offficers for the ensuing year:—President, Louis J. Hirschman, M. D., Detroit, Mich.; Vice-President, Alois B. Graham, M. D., Indianapolis, Ind.; Secretary-Treasurer, Lewis H. Adler, Jr., M. D., Philadelphia, Pa.

Executive Council.—John L. Jelks, M. D., Memphis Tenn.; Louis J. Hirschman, M. D., Chicago, Ill.; J. Rawson Pennington, M. D., Chicago, Ill.; Lewis H. Adler, M. D., Philadelphia, Pa.

The place of meeting for 1913 will be at Minneapolis, Minn.

Exact date and headquarters to be announced later.

The following were elected Associate Fellows of the Society:

Dr. Rollin H. Barnes, Metropolitan Building, St. Louis, Mo.; Dr. Zarney J. Dryfuss, 7 W. 91st St., New York City, N. Y.; Dr. James A. Duncan, 1107 Broadway, Toledo, O.

---

The following is an abstract of the principal papers read:

### PRESIDENT'S ADDRESS.

### RELATIONSHIP AND DUTIES OF "THE PROCTOLOGIST" TO THE PROFESSION.

BY JOHN L. JELKS, M. D., OF MEMPHIS, TENN.

He stated that this Society was an innovation when organized—a strange vessel on the high seas. A child of American

Medicine, it has now become a sprightly youth, with ambition and strength of purpose, having and exercising authority.

The medical world recognizes as authoritive, the expressions of its Fellows in the field covered.

He admonished discretion thorough description and perfection of technic. Hasty speech or carelessly written papers cannot be erased or changed—as in their publication they become a permanent record.

He referred to the theories of our science, which were born of dreamers and nurtured by enthusiasts, and fancies no solid superstructure could be reared on foundations so infirm, and added that neither these, nor the honor, distinction, nor the gain they held out, should be sufficient to determine the surgeon to make merchandise of theories.

He called attention to the obstacles this Society had encounered, because of these fragile theories, which had, previous to its existence, been set up as targets for those who were unfavorable to the development and progress of this specialty.

He considered the true surgeon and specialist as humanitarian, whose purpose in life is to save life, restore health and happiness, and admonished him to shield and protect his brother from the darts aimed to destroy.

He also referred to cancer in the rectum, sigmoid and colon, which may have been treated as of minor significance until metastases are so extensive as to preclude hope of a cure. He praised those Proctologists, who have with much patience and fortitude, labored for and finally have overthrown that unfortunate assignment of malignant rectal and colonic cases to untimely graves.

He stated that much harm has been done by the profession in the establishment of drug habits among the American people for the relief of constipation as last year's symposium before this Society would show, and says the Proctologist is best equipped to study these cases, and arrive at the true etiology pointing to means of relief.

The American people are living in tin cans and cracker boxes, sparing time only to catch the next train, or meet the next market report, are storming their nervous systems with destructive toxins, filling sanatoria and health resorts with wrecks and lowering the scale of human usefulness and intelligence. None can more early observe the impending catastrophe, or turn on the search-light as the Procto-Enterologist, and scientist, who calls together the aids of chemistry, physiology, pathology and bacteriology and a fair degree of understanding as to the results of the methods and habits of life of the average American citizen, who is less careful in the selection and preparation of his own food than that of his stock.

He complimented the Fellowship of the Society, which is limited to fifty and has forty-three members, and stated no similar number of men are banded together in the civilized world who can boast of greater attainments for the science of medicine, or for humanity, almost every member being the author, or an associate author of a book, and these are all standard text or reference books in this branch, most of them also have been inventors of valuable instruments, or appliances applicable to this specialty.

He referred to some of the research work done by the Fellows, and to the possibilities yet before them in Procto-Enterology.

He alluded to the intra- and extra-rectal and anal colonic infections, the role they play and the possible developments of vaccine therapy and antitoxins in combating them. He stated that each Fellow should carefully weigh his selected subject for these meeings, being mindful of the fact that the general profession is looking to this Society and its individual Fellows for facts, not fancies; for proven remedies and technics, and not fads.

The Society has attained an individuality, both national and international, and he reminded his Fellows that there is labor yet to perform. That they must retain their progressive spirit and enthusiasm, lest they lapse into a state of self-satisfaction when retrogression will mean their ending.

He referred to the fact that few of the hospitals of this country permit additions to their staff of specialists in Proctologic work, hence the general surgeon and the general practitioner are doing the work in these institutions, about as these same men would do the Ophthalmologic work, etc.

He recommended the addition to the American Medical Association of a section, in which the subjects Gastro-Enterology and Proctology, or Procto-Enterology may be discussed.

He advised closer confinement of the Proctologists to this work, to the exclusion of general work, and believed this will recive from the profession greater respect for this specialty. and that fewer of this class of cases will be referred the general surgeon, or be accepted by him for treatment.

Conservative Life Insurance Companies are now convinced of the necessity of paying attention to the rectum and colon and such instances as the writer's confidential reports to alert examiners of cases of Amebic infection, Adenomata, Papillomata, syphilitic or tuberculous diseases, which the examiner would have overlooked, and impressed him with this fact, and he wondered if these and similar instances had not brought to the minds of medical referees the possible advisability of subjecting all applicants for large policies to a plurality of examiners. He advised the change of name of this society to that of The American Procto-Enterologic Society, and stated not one of the Fellows of the Society had found he could eliminate from his work intra-abdominal intestinal work.

---

*A Review of Proctologic Literature for* 1911.—*By Samuel T. Earle, M. D., of Baltimore, M. D., Chairman of the Committee on same.*

Dr. Joseph F. Saphir, of New York City, the New York Medical Journal, 1911, Vol. 93, page 216, gives a description of "A Syringe for Local Anaesthesia in Rectal Operations."

Von Dr. Erich Schlesinger, Berlin, Deutsche Medizinische Wochenschrift, February 9, 1911, report "An Air Pessary for Keeping in Place Internal Hemorrhoids and Prolapse of the Rectum."

Dr. Thomas B. Noble, Indianapolis, Ind., American Journal of Obstetrics, 1910 Vol. 61, page 259, has devised an instrument known as the "Anastomat," to facilitate the end-to-end anastomsis inextirpation of the rectum and sigmoid.

Dr. Dudley Roberts, Brooklyn, N. Y., The Proctologist, 1910, Vol. 4 gives a description of "A New Anal Speculum."

Dr. James F. Churchill, Chicago, Ill., Surgery, Gynecology and Obstetrics, Vol. XI, 1911, page 205, gives an interesting paper on "Rectal Anaesthesia."

Leslie W. Dryland M. R. C. S. England, L. R C. P. London, D. P. H. London Lancet, 1910, Vol. II, page 810. "An Operation for Prolapse of the Rectum."

Sidney Boyd replies to the above paper of Leslie Dryland's London Lancet, 1910, Vol. II, page 1242.

Dr. L. L. McArthur, Chicago, Ill., Journal of American Medical Association, 1911, Vol. LVII, page 363. "Rectal Prolapse."

Dr. Kenneth A. J. McKenzie, Portland, Ore. Transactions of the American Surgical Association, 1911, Surgery, Gynecology and Obstetrics, 1911 Vol. 13, page 218. "Treatment of Fistula in Ano without Mutilation of the Sphincters."

A Campbell Magarey, M. B., Adelaide, M. R. C. S., England. British Medical Journal, 1911, Vol. 2, page 71. "Hypertrophied Papillae of Morgagni."

---

### CRAWFORD W. LONG.

By John Chalmers Da Costa, M. D., LL. D., Gross Professor of Surgery in Jefferson Medical College.

*(Continued from Last Issue.)*

We may conclude that when Long left this school he understood the agony caused by surgery and realized what a great thing it would be to be able to operate without causing pain, that he had no belief in the value of "animal magnetism," as a surgical anaesthetic—that he knew that nitrous oxide, when inhaled, would produce delirium—that he knew that ether inhalations were given therapeutically and sometimes taken for sport, and that large doses would produce unconsciousness. He had been taught, and probably at that time believed, that only small doses were admissible and that doses large enough to produce unconsciousness would bring deadly peril to the patient. He likewise took with him the council of Wood regarding the necessity of being ever cautious in reputing results.

After graduation he went to New York City and "walked the hospitals." In that city he had the opportunity to hear Valentine Mott, J. Kearney Rogers and Willard Parker. He wished to enter the medical corps of the U. S. Army but his father vetoed the plan, so he returned to Georgia in 1841, and began general practice in Jefferson, a village in Jackson County.

The year 1841 was the very year that Esdaile, in India, performed so many operations upon hypnotized subjects, that Braid, of Manchester, began to set forth his views on induced trance, and that Elliotson began to warmly advocate hypnotism as a surgical anaesthetic.

Here is the story of Long's discovery in his own words*—"In the month of December, 1841, or in January, 1842, the subject of inhalation of nitrous oxide gas was introduced in a company of young men in this village; several persons present desired me to produce some for their use. I informed them that I had no apparatus for preparing or preserving the gas, but that I had a medicine (sulphuric ether) which would produce equally exhilarating effects: that I had inhaled it myself, and considered it as safe as the nitrous oxide gas. One of the company stated that he had inhaled ether while at school, and was then willing to inhale it. The company were all anxious to witness its effects. The ether was introduced. I gave it first to the gentleman who had previously inhaled it, then inhaled it myself, and afterwards gave it to all persons present. They were so much pleased with the exhilarating effects of ether, that they afterwards inhaled it freely and induced others to do so, and its inhalation now became fashionable in this country, and, in fact, extended from this place through several counties in this part of Georgia."

We may note that R. H. Goodman, one of the persons who participated in an ether frolic in Jefferson, made an affidavit in 1853, stating this fact and also that he removed to Athens, January 20, 1842, and introduced ether frolics in that community. It is interesting to observe that Long had inhaled ether before the first ether frolic, and that, repudiating the teaching he had received as a student, he regarded it as being as safe as nitrous oxide. To continue Dr. Long's narrative: "On numerous occasions I have inhaled ether for its exhilarating properties, and would frequently, at some short time subsequent to its inhalation, discover bruises or painful spots on my person, which I had received while under the influence of ether. I noticed my friends, while etherized, received falls and bangs which I believed were sufficient to produce pain on a person not in a state of anaesthesia, and on questioning them, they uniformly assured me that they did not feel the least pain from these accidents. These facts are mentioned that the reasons may be apparent why I was induced to make an experiment in etherization.

"The first patient to whom I administered ether in a surgical operation was Mr. James M. Venable, who then resided within two miles of Jefferson, and at present (1849) lives in Cobb County, Georgia. Mr. Venable consulted me on several occasions in regard to the propriety of removing two small tumors situated on the back of his neck, but would postpone, from time to time, having the operations performed, from dread of pain. At length I mentioned to him the fact of my receiving bruises while under the influence of the vapour of ether, without suffering, and as I knew him to be fond of and accustomed to inhale ether, I suggested to him the probability that the operations might be performed without pain, and proposed operating on him while under its influence. He consented to have one tumor removed, and the operation was performed the same evening. The ether was given to Mr. Venable on a towel, and when fully under its influence, I extirpated the tumor. It was encysted and about one-half inch in diameter. The patient continued to inhale ether during the time of operation, and when informed it was over, seemed incredulous, until the tumor was shown him. He gave no evidence of suffering during the operation, and assured me after it was over that he did not experience the slightest degree of pain from its performance. This operation was performed on March 30, 1842."

When Long finished that operation he must have felt a sense of combined wonder, exultation and responsibility. It was a brave thing to operate under the full influence of a drug when all professional teaching was that it required large amounts of the vapour to produce unconsciousness, and that large amounts were dangerous. Had the patient died, the doctor would have had a lifelong self-reproach and would possibly have been sued or prosecuted for manslaughter.* It was brave of Venable to take the chance. Wonder would naturally arise in Long's mind as he thought of the agonies inflicted by the surgery he had seen in Philadelphia and New York, as compared with the perfect tranquility of the patient just operated upon. Exultation would be inseparable from the accomplishment of what the masters of surgery regarded as impossible. A sense of grave responsibility would be in a man who believed he had done a mighty thing, but felt the necessity of proving it thoroughly in order that he might not mislead others and do harm.

He saw the beneficent light break into the dark dungeons of pain. He must have felt as did Sinbad, the Sailor, when, from the living tomb in which he was immured, he saw the glad rays of the sun. He and his companions might well have exclaimed with the ancient Mariner:

"We were the first that ever burst, into that silent sea."

That is the story of the first use of ether inhalation to still the pains of surgery.

What of the personality, the character, of the man who discovered anaesthesia? In August, 1842, he married Mary C. Swain. It was a peculiarly happy union. His wife was an intellectual woman and a thoroughly congenial helpmate. She was the inspiration of his life. She fitted herself to understand and sympathize with all his wants and needs. They were real lovers when they married and remained lovers until death parted them. He remained a resident of Jefferson until 1851, when he removed to Athens, Georgia. He lived in Athens until his death, in 1878, and practiced there continuously except during his service in the Confederate Army.

For nearly thirty years he was in very active service, was in the habit of riding miles through the country, and endured all the hardships of a busy country practitioner. No man was ever loved more. All his patients were his devoted admirers. His personality impressed itself upon them. He was counselor and friend as well as physician. He always placed the welfare of his fellows before his own. He was more than a great man, he was a good man. He was one of those rare individuals who really practice their religion. The words of his faith were not mere empty formulas, as with so many, but were mandates to fine deeds. He carried with him through life no ignoble rancor. Disappointment there must have been but there was never hatred of his fellows. He had been excluded from honors that were justly his, but he never kept the thought of it as "something bitter to chew on when feeling Byronic." He in no sense became that desolating human calamity, an embodied grievance. A grievance wears out sympathies and tires out our appreciation. There was nothing morbid in his temperament. He never scoffed at Destiny or denounced Fate. He never claimed to be an unappreciated spirit or a misunderstood soul. He calmly went his useful way, tending the sick, aiding the needy, caring for his own, sure of himself, confident of the future, never boasting, never brooding, kindly and fair to all, generous ever to opponents, courteous even to critics, and making no struggle for stained wreaths or for tarnished rewards. He was a complete man, a rounded character, a true physician, and when we honor him we find no apologies necessary. He never tried to patent and thus coin into dollars a discovery which has brought and will bring comfort unspeakable to countless thousands of the race. He thoroughly loved his profession. He said: "I am as much called to practice medicine as a minister is to preach the gospel!"

He accepted all medical tasks as commands which he was glad to be thought worthy to receive and fit to execute.

He had that splendid combination, strength and tenderness. He inspired trust. Surely he must have done so, else Venable would never have taken ether to unconsciousness. He was wise and self-confident, else he could never have given Venable ether to unconsciousness when all the leaders of medicine taught that such doses were highly dangerous.

He was full of sympathy for suffering and cared for the lowliest as for the richest. He was gentle, forbearing, faithful to every duty and every instinct. He was always dignified and usually reserved, relaxing at times into gaiety in his family circle among those who knew him well. He had a vein of humor, was given to jests when by his own fireside, and now and then sent humorous sketches to the local newspapers. He was simple of heart, and pure in word and act.

He was a close observer; a hard worker; was honest in thought, word and deed—hated all lies and anything that even savored of deception. His life was lived in the light of day without any stratagems or pretenses. He was straightforward and unsuspicious, hated to hold ill opinions of anyone, and only a native ability to judge character saved him from frequent impositions. His family adored him. He liked to read aloud to his children and brought them up on the works of Scott, Dickens, Shakespeare and other master minds. He was particularly fond of Hamlet. At bed time he followed the old time custom of reading the Bible to the assembled family. He was fond of whist and was one of the best of players. He was devoted to farming, was a good business man and an excellent executive.

In slavery days he was, as were most Southern gentlemen, a kind master to his slaves. He believed that slavery was a plan of Providence to civilize the negroes. He thought that to own slaves was a great and terrible personal responsibility, a responsibility which he ranked close after the one owed to his wife and children. In an old journal he writes: "God grant that I may be a tenderer husband and father and a better master." When his slaves had become free he still watched over their welfare, cared for them when sick, relieved their necessities and gave them useful counsel. The blacks loved and trusted him as much as did the whites.

He had a great reverence for womanhood. He would carry a basket for the lowliest woman with the courtly air others might show to a princess. A veritable termagent used to haul wood into town to sell. Again and again when he met her he bought the load and took it to his own house. On one occasion Mrs. Long said to him: "We have plenty of wood, why do you always buy that woman's," and he said, "because I hate people to see a woman doing man's work." He would go any distance and attend the poorest negress in labor because of his sympathy for those in the pains of childbirth and his reverence for maternity.

At the unveiling of the monument in Jefferson, Dr. Woods Hutchinson said that Long was in many respects in advance of his day—that he treated and cured consumption by food, fresh air and tonics—that he treated typhoid fever practically as we do now—that he treated that very dangerous disease bilious fever by quinine when few did so—and that he operated many times very successfully for cancer of the breast, always clearing the ribs and removing the axillary glands. (Munsey, August, 1911.)

It is interesting to note that he never charged more than one hundred dollars for a "breast operation," even if the patient was very well off.

He was a Whig in politics and strongly opposed to secession. When Georgia resolved to go out of the Union Long said: "This is the saddest day of my life." Naturally he stood with his own people and went with his state. He entered the Confederate army and served through the war. Like all his friends he lost everything by the war, and he suffered along with them the horrors of reconstruction and the infamous tyrannies of carpet-bag rule. Soon after the war Long was offered the position of United States Contract Surgeon to help care for the many sick and wounded soldiers in Georgia. He was not even asked to take the oath of allegiance. The fifty dollars a month, paid him for his work, came as a blessing in those dark days of poverty. After some years he again became prosperous.

Once, when his health was impaired by over-work, his friends and family urged him to take a holiday, but he said ,"my sick need me."

In his 63rd year he was struck by apoplexy when at a sick woman's bedside. The moment he recovered consciousness he asked how she was. Before he passed into the unconsciousness which was to end in the long sleep and the silent house, he gave direction for the sick woman's care. He was faithful to duty to the last. He died June 16, 1878.

Such was Crawford W. Long. The University of Pennsylvania this day hangs his likeness in the Hall of Fame with her noblest sons. He was an honor to his alma mater, an ornament to his profession, a glory to his country, and a benefactor to the human race.

REPLY BY HON. SAMUEL J. TRIBBLE.

At the conclusion of the ceremonies, Hon. Samuel J. Tribble United States Congressman from Georgia, spoke as follows:

In behalf of the family of Dr. Long and the State of Georgia, I thank you, Mr. Provost, for the honor conferred by the University of Pennsylvania on the distinguished son of my State. This tribute to a great man with no military or political renown is a high testimonial of the progressive thought of this University. History loves to honor the hero. The boy, the man, yea, the woman, all are hero worshipers. Alexander the Great led his armies into all known countries and humanity will never tire of reading his achievements as the Conqueror of the World. Napoleon scaled the Alps and laid waste the plains of Italy, and we read, with charm, volume after volume in history and fiction of the gratest military genius the world has ever known. These and other military heroes left devastated fields, desolation and want, widows and orphans, pain, sorrow and death written on the pages of history. To-day you erect a Long Medallion and commemorate the memory of a man who carried no military trophies to his grave, made no widows and devastated no fields, but sir, he alleviated the pains of humanity throughout the earth. The Great Teacher—our Master—taught that the greatness of men should be measured by the good they do. Applying this mould to Dr. Long, he becomes one of the greatest men of modern or ancient times.

In his native village in Georgia there has been erected a marble shaft to his memory; to the foot of that shaft our children, for generations will go and point to the name of this great Georgia humanitarian; to the University of Pennsylvania your childrens' children will come and point to the name on this tablet erected by you, as one of the greatest men of the Nation; to the Capitol of the Country at Washington, where his statue will be placed in the Hall of Fame, citizens of foreign countries will come and point to his name on the statue in that Hall as one of the greatest men the world ever knew. Georgia, his home, and Pennsylvania, his Alma Mater State, strike hands to-day to do him honor, and when his statue is erected at the National Capitol, the whole Nation will join us in the memorial, and his greatness will be glory enough for all.

Dr. Long comes from a section of statemen. In the radius of a few miles, if time permitted, I could point you to Wm. H. Crawford, who ranked with Calhoun, Clay and Webster, and needed only one more vote to give Georgia a President; I could point you Alexander H. Stevens, the greatest statesman the South ever produced; I could point you Robert Toombs, one of the greatest minds and orators of the Union; I could point you to the Cobbs, statesmen of the Webster and Clay type; I could point you to Benj. H. Hill, who stood, in the breach of reconstruction days, on the floor of Congress, eyes flaming with defiance, and yet rising above his sectional animosity, and uttering such speeches as "we felt your heavy arms in the carnage of battle, and above the roar of the cannon we heard your voice calling, brothers, come back;" I could point you to Henry W. Grady, bearing an olive branch, and with his matchless eloquence, wiping out sectional animosity in every section of the country. These, and many others; but, Mr. Provost, last but not least of this array of greatness, I point you to Crawford W. Long, not a statesman, not a war hero, but the alleviator of human pain the world over.

